# Next generation sequencing and analysis of a conserved transcriptome of New Zealand's kiwi

**DOI:** 10.1186/1471-2148-10-387

**Published:** 2010-12-15

**Authors:** Sankar Subramanian, Leon Huynen, Craig D Millar, David M Lambert

**Affiliations:** 1 Griffith School of Environment and the School of Biomolecular and Physical Sciences, Griffith University, 170 Kessels Road, Nathan, Qld 4111 Australia; 2 Allan Wilson Centre for Molecular Ecology and Evolution, Institute of Molecular BioSciences, Massey University, Auckland, New Zealand; 3 Allan Wilson Centre for Molecular Ecology and Evolution, School of Biological Sciences, University of Auckland, Private Bag 92019, Auckland, New Zealand

## Abstract

**Background:**

Kiwi is a highly distinctive, flightless and endangered ratite bird endemic to New Zealand. To understand the patterns of molecular evolution of the nuclear protein-coding genes in brown kiwi (*Apteryx australis mantelli*) and to determine the timescale of avian history we sequenced a transcriptome obtained from a kiwi embryo using next generation sequencing methods. We then assembled the conserved protein-coding regions using the chicken proteome as a scaffold.

**Results:**

Using 1,543 conserved protein coding genes we estimated the neutral evolutionary divergence between the kiwi and chicken to be ~45%, which is approximately equal to the divergence computed for the human-mouse pair using the same set of genes. A large fraction of genes was found to be under high selective constraint, as most of the expressed genes appeared to be involved in developmental gene regulation. Our study suggests a significant relationship between gene expression levels and protein evolution. Using sequences from over 700 nuclear genes we estimated the divergence between the two basal avian groups, Palaeognathae and Neognathae to be 132 million years, which is consistent with previous studies using mitochondrial genes.

**Conclusions:**

The results of this investigation revealed patterns of mutation and purifying selection in conserved protein coding regions in birds. Furthermore this study suggests a relatively cost-effective way of obtaining a glimpse into the fundamental molecular evolutionary attributes of a genome, particularly when no closely related genomic sequence is available.

## Background

DNA sequencing technologies have enabled us to decipher molecular sequences of individual organisms. Conventional DNA sequencing methods relying on fluorescent dideoxy terminators and capillary separation revolutionized sequencing and allowed the first constructions of complete genomes of a number of species from simple prokaryotes to higher vertebrates [1,2]. However the costs involved in eukaryotic genome-sequencing projects using these methods has been very high and thus such projects generally require the collaboration of several well-funded institutes. Furthermore the time required for sequencing and assembling such genomes can span several years. The advent of Next Generation DNA sequencing has reduced the time and expense of complete genome sequencing by orders of magnitude [3,4]. For example using next generation sequencing methods the complete genome of a human individual was completed in eight weeks by spending only a fraction of cost incurred using conventional BAC and shotgun-based cloning and sequencing methods [5].

The major limitation of a next generation sequencing approach is that the length of the sequence reads produced was until recently only 25-200 bases, as opposed to over a kilobase generated by conventional capillary-based sequencing methods. Although short sequence reads do not limit the amount of sequence data collected, this can hamper the assembly of the short sequence reads into large contigs. Therefore the availability of a genome of a closely related organism is generally required (to act as a scaffold) for the successful assembly of a new genome using the next generation sequencing method. For example, the assembly of the genomes of Neanderthal and Woolly Mammoth was possible only because of the availability, respectively, of complete human and African elephant genomes [6,7].

Recently new algorithms have been developed to assemble genomes *de novo *without using a closely related scaffold genome [8]. However for precise genome assembly using this software, substantial sequence coverage is required. For example, using next generation sequencing and *de novo *assembly the complete genome of a Chinese panda was obtained at 50× (times) coverage [9]. Although the sequence required (150 gigabases) was generated in only one month, the cost amounted to several million US dollars. Therefore without the availability of a closely related organism, assembling complete genomes *de novo *would still be financially restrictive for most research laboratories. An alternative would be to use low-coverage next generation sequencing to sequence and assemble only the transcribed regions of a genome (transcriptome).

Large-scale comparative genomic studies of avian genomes started soon after the chicken genome became available [10]. These studies revealed mutational rate differences among chromosomes by comparative analyses of chicken and turkey genomes [11,12]. With the availability of the complete genome of the zebra finch [13] a number of studies have examined genome-wide patterns of molecular evolution and gene expression in avian genomes [14-17]. However all these studies were performed using only the Neognathae birds.

In the current study we have sequenced and assembled a number of conserved protein-coding regions of an early stage transcriptome of a Paleognathae bird, the North Island Brown kiwi (*Apteryx australis mantelli*). Kiwi are flightless birds endemic to New Zealand, and are of high conservation importance. The purpose of this study was to isolate conserved transcribed sequences of kiwi in order to understand firstly the patterns of mutation and selection amongst protein coding sequences and secondly to use these nuclear gene sequences to reanalyze the divergence time between Paleo- and Neognathae birds.

## Results and Discussion

### Preliminary kiwi transcriptome analysis

Initial cDNA made from kiwi embryo k11-15 was tested for coverage by amplification of the temporally and spatially restricted developmental genes *tbx5*, *cry1*, *pax6*, *BMP4*, *ptx1*, *hoxB1*, *hoxB8*, and *hoxD12*. All genes were successfully amplified from the kiwi cDNA suggesting a comprehensive early stage kiwi transcriptome. As expected, comparison of the amplified kiwi gene sequences with those in GenBank consistently gave the chicken homologue as the closest match.

### Assembly of the conserved regions of Kiwi protein-coding genes

**Figure 1:**
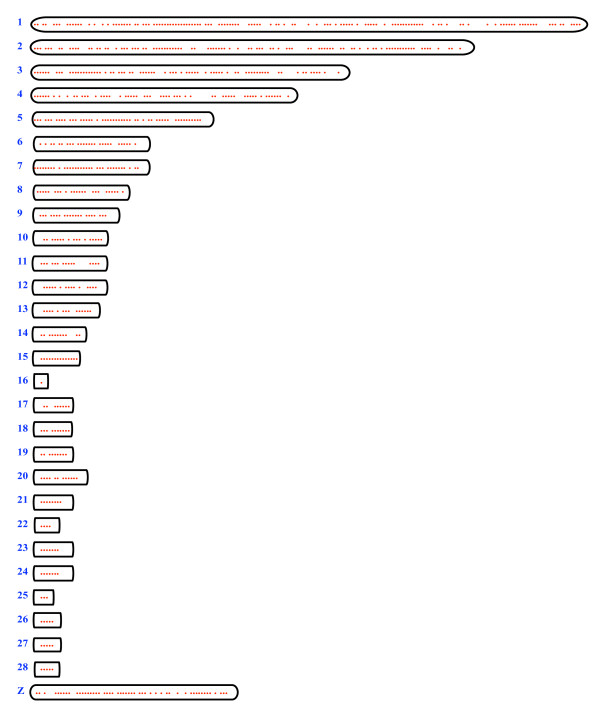
**Chromosomal locations of orthologous kiwi genes recovered in this study on the chicken genome**.

**Figure 2:**
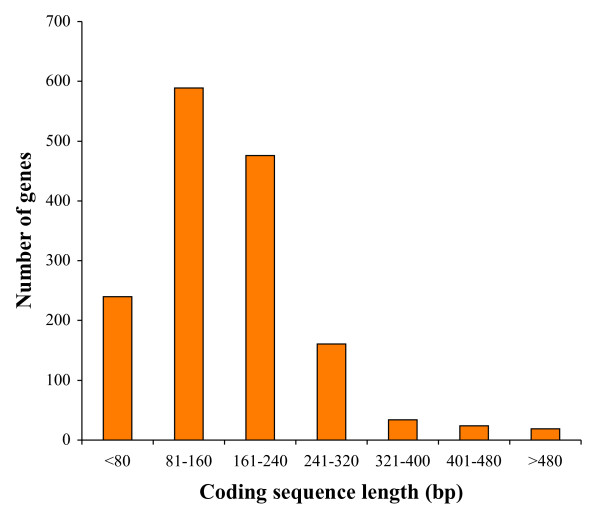
**The distribution of the lengths of kiwi protein-coding regions sequenced and assembled in this study**.

To assemble over 75,000 FLX sequence reads we used the well-annotated chicken proteome. Kiwi sequences were used as a query to search over 22,000 chicken proteins using BLASTX. Significant hits were garnered and redundant kiwi reads were excluded. Garnered kiwi sequences were assembled into large contiguous segments. We used Blast2seq to align chicken proteins with translated assembled reads of kiwi. Finally we retained only the regions of the assembled reads where at least 90% of the amino acids aligned with their respective chicken orthologs. Although this resulted in a significant reduction in the number of kiwi genes retained, such an approach was required to minimize lineage specific duplicates. Furthermore we are confident that this approach will exclude most of the transcribed pseudogenes, which generally have a high rate of substitution. This stringent approach identified 1,543 kiwi genes. The locations of the chicken-orthologs of kiwi genes on the chicken chromosomes are shown in Figure 1. The average length of coding regions was 168 bp (Figure 2). Our approach to assemble the transcriptome is somewhat similar to a previous method, SCARF [18]. However SCARF is suitable only if the reference scaffold genome is closely related to the target genome with a synonymous divergence of <0.1.

### Embryonic expression levels of kiwi genes

**Figure 3:**
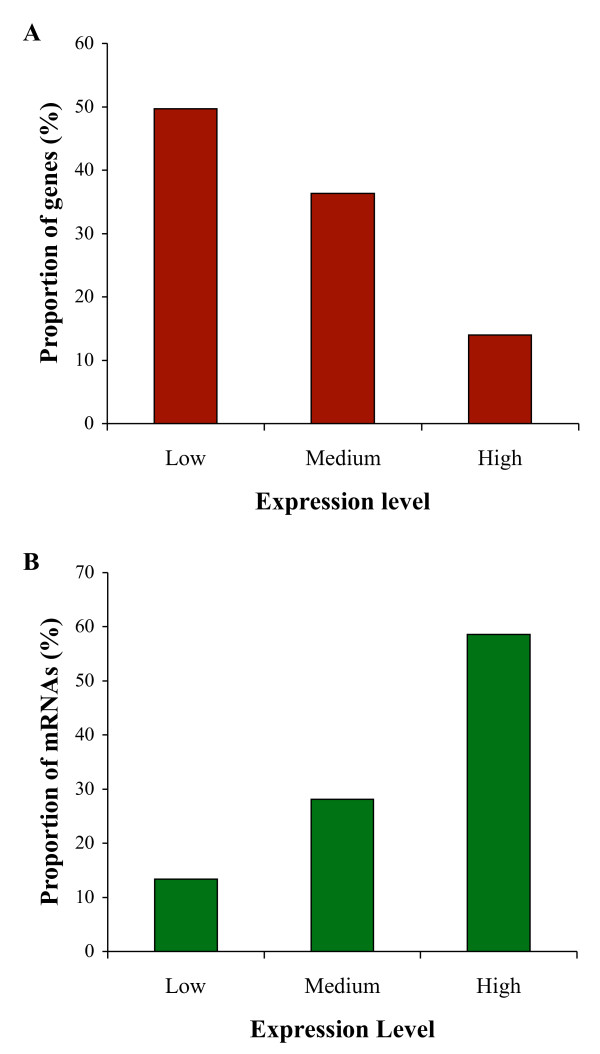
**Kiwi transcript expression levels**. **A**. Proportion of kiwi genes belonging to high (> 5 copies), medium (2-5 copies) and low (1 copy) expression levels based on the number of mRNA copies. **B**. Percentage of mRNAs belonging to different expression level categories.

Since the sequences generated in this study were from the mRNAs of a kiwi embryo, the copy numbers of the genes reflect their expression levels. We found two genes with very high (> 2000 copies) expression levels. While we identified (through the functional annotation of its chicken ortholog) the product of one of these genes as a neuropeptide the function of the other could not be determined. We grouped kiwi genes into three categories based on their levels of expression. Roughly 14% of the genes were highly expressed (> 5 copies) while 50% of the genes appeared to be present as single copies in kiwi embryo (Figure 3A). Conversely the small fraction of highly expressed genes constituted approximately 59% of the expressed mRNAs in the embryo, whereas only 13% of the mRNAs were from genes with low expression levels (Figure 3B). For this analysis we did not include the two genes with very high expression levels (mentioned above) to avoid bias due to these outliers. Gene annotations show that most of the highly expressed genes were those involved in protein synthesis such as ribosomal proteins, elongation factors, tRNA synthetases, as well as structural proteins such as collagen.

### Evolutionary divergence at neutral and constrained sites

An important parameter used extensively in genome analyses is the rate of molecular evolution. To examine the rate of neutral evolution we compared the concatenated kiwi transcripts with those of chicken. A likelihood-based distance analysis showed that divergence at neutral synonymous sites was 0.465 substitutions per site. Conversely, divergence at nonsynonymous positions was only 0.071 substitutions per site; over an order of magnitude less than that of neutral divergence and suggests that high levels of purifying selection are acting on amino acid replacement sites. This is perhaps not surprising as the genes analyzed in this study are expressed early in embryonic development and therefore most are expected to be highly conserved. The average ratio of nonsynonymous- to synonymous divergences (dN/dS) was 0.15 (± 0.002), which is comparable to that estimated using chicken-zebra finch sequences for the genes expressed in zebra finch embryo [16]. A previous study using zebra finch and emu (another palaeognathae bird) estimated the divergences at synonymous and nonsynonymous positions to be 0.47 and 0.04 respectively [19]. However we found a much higher divergence between zebra finch and kiwi at synonymous (0.53) and nonsynonymous (0.07) positions, which suggests that the rate of evolution in kiwi might be faster than that of emu.

**Figure 4:**
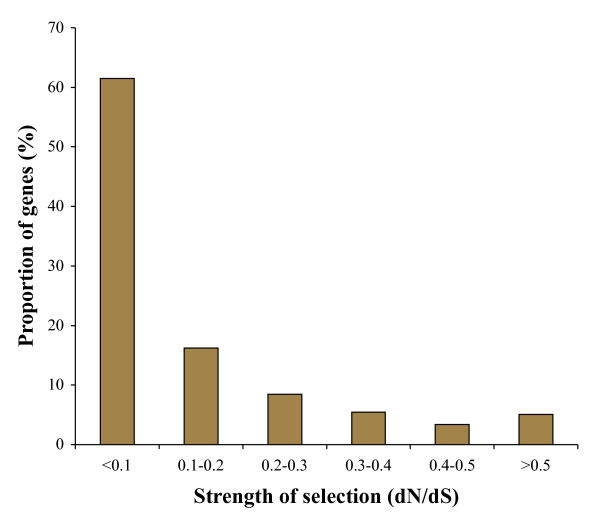
**Distribution of kiwi genes under varying levels of selective constraint**. The strength of selection was determined by the ratio of divergence at nonsynoymous positions (dN) to the divergence at synonymous sites (dS). The divergence (substitutions per site) was estimated for 1,543 kiwi-chicken orthologus gene pairs using PAML [31].

The distribution of the dN/dS ratio estimated for individual genes shows that over 60% of the genes were highly constrained (dN/dS < 0.1) (Figure 4). On the other hand only 5% of the genes had a high (> 0.5) dN/dS ratio. Among these we found 25 genes that appear to be evolving under positive selection (dN/dS > 1.0). However the difference between the dN and dS estimates was not statistically different (higher) for any gene. This is primarily due to the high variance in the estimates, caused by the short sequence length of the partial genes used in this analysis and this adversely affects dS estimates over dN estimates, as synonymous sites are fewer than replacement sites. A previous study observed a higher rate of evolution in the zebra finch lineage compared to chicken using anole lizard as an outgroup [17]. We examined this using kiwi as an outgroup and found an approximately 50% higher rate of evolution in the zebra finch lineage compared to chicken at synonymous (49%) as well as at nonsynonymous (57%) positions.

To compare sequence divergence between the birds and mammals we obtained the orthologous human and mouse genes and used the alignment that contained only the aligned regions from kiwi, chicken, human and mouse. Therefore we could examine the rates and patterns of evolution in the same genes and regions from mammals and birds. This analysis using 616 genes showed that the divergence at synonymous sites between chicken and kiwi was almost identical to that between human and mouse (0.453 Vs 0.465, *P *= 0.13). Therefore the mutation rates of mammals and birds could be similar if the divergence times between human-mouse and chicken-kiwi splits are comparable. Molecular data based studies estimates suggest a ~115 My split for primates-rodents divergence [20] and a ~130 My split for paleo-neognathae birds [21,22]. Furthermore fossil-based estimates also suggest a similar divergence time for the primates-rodents split (62 My - 100 My) and for palaeognathae-neognathae split [23]. However the nonsynonymous divergence for the chicken-kiwi pair was significantly higher than that estimated for the human-mouse comparison (0.055 Vs 0.028, *P *< 0.0001). This suggests that despite the similarity in neutral substitution rates between mammals and birds, the magnitude of purifying selection appears to be much higher in the former than the latter. Although the low coverage of our sequence data might include some sequencing errors (< 0.005 per site) it will not significantly affect our results as the comparative analyses presented here involve only distantly related species.

**Figure 5:**
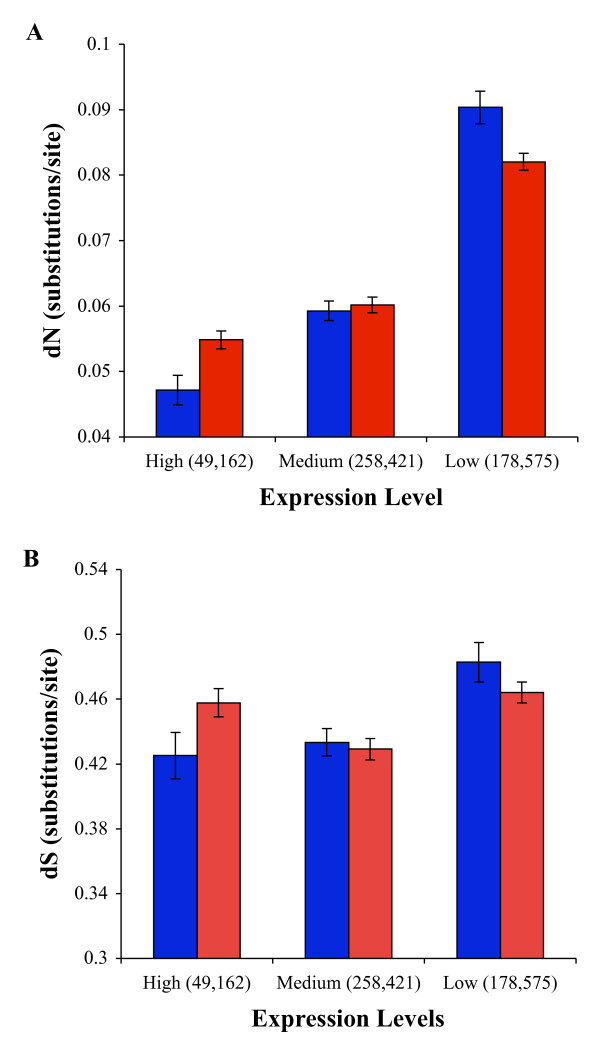
**Rate of evolution of conserved kiwi coding sequences and gene expression levels**. The three categories of expression level are based on kiwi as well as chicken embryonic expression data (blue) or based on kiwi embryonic expression data only (red). **A**. Mean divergence (substitutions per site) at nonsynonymous sites in kiwi genes from different expression levels. **B**. Average divergence at synonymous positions. Error bars show the standard error of the mean estimates. The number of genes used are given in parentheses.

Previous studies showed a low nonsynonymous divergence in highly expressed genes, owing to a higher selection constraint on these genes [24,25]. We examined this for the kiwi-chicken species pair and found that this also holds true for these avian species. We found that the highly expressed genes are generally highly constrained and conversely the rate of evolution at nonsynonymous sites of the genes with low expression levels showed a large variation [see also [25]]. Therefore a simple correlation analysis does not capture the actual relationship between expression level and the rate of nonsynonymous site evolution. Hence, we estimated the divergences at synonymous and nonsynonymous sites of genes with high, medium and low expression levels by concatenating the genes in each group. The nonsynonymous divergence of genes with low expression levels was found to be 50% higher than that of the genes with a high expression level (0.082 vs 0.054) and this difference was statistically significant (*P *< 0.0001) using a Z-test (Figure 5A, red). However the divergence at synonymous sites of lowly expressed genes was not significantly different to that of the highly expressed genes (0.464 vs 0.458 *P *= 0.58) (Figure 5B, red).

The expression levels determined for kiwi genes might be influenced by the level of coverage of individual genes. Therefore we obtained the expression levels for the orthologous chicken genes using an embryonic chicken library (with 22000 ESTs) [26]. Only those genes that had a high expression level (> 5 copies of mRNA) in kiwi as well as in chicken embryos were designated as highly expressed genes. Similarly we determined the genes with medium and low expression levels. Although this reduced the number of genes in our analysis, this stringent approach also produced similar relationships between expression levels and the divergences (Figure 5A and 5B, blue). This analysis showed an almost two fold higher nonsynonymous divergence for lowly expressed genes than that of the genes with high expression level (0.090 Vs 0.047, *P *< 0.0001). The difference in the synonymous divergences between the genes with low and high expression levels was only 14% but was statistically significant (0.483 Vs 0.425, *P *= 0.002). This result suggests very weak selection in synonymous sites of conserved genes of birds.

### Functional categories of proteins expressed in the kiwi embryo

**Figure 6:**
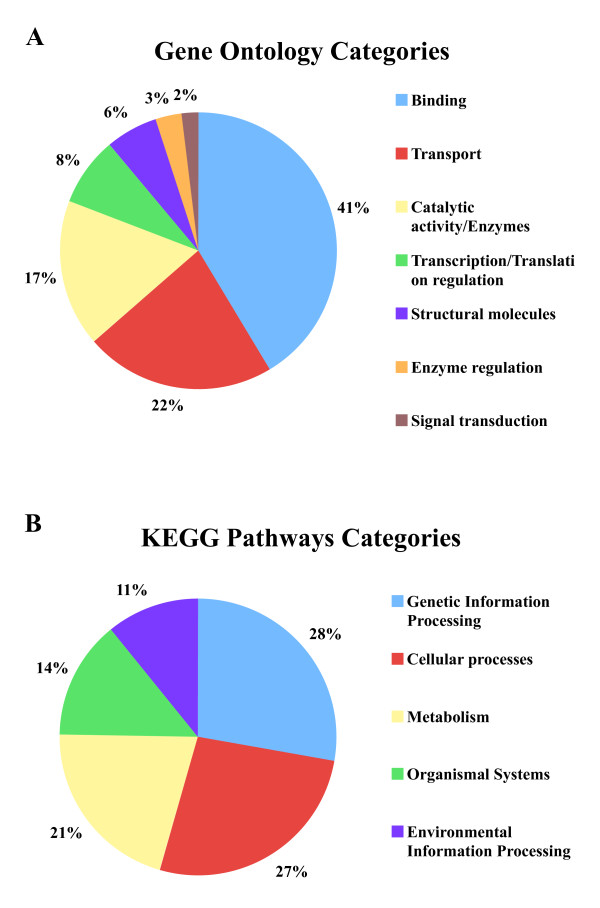
**Functional categories of kiwi genes**. **A**. Functional classifications are as described in the Gene Ontology Consortium http://www.geneontology.org. **B**. Classification of genes are based on their involvement in various pathways as categorized by the KEGG pathways database http://www.genome.jp/kegg/pathway.html. The functions and pathway involvement of kiwi genes were deduced from their chicken counterparts for which gene ontology functions or KEGG pathway information were available.

To determine the functions associated with the kiwi proteins we obtained the functional annotations of their orthologous chicken counterparts from the Biomart resource ENSEMBL http://www.ensembl.org. Our search using the gene ontology classification of chicken genes resulted in the identification of gene function for ~500 genes. We found that the majority of the kiwi genes (41%) code for DNA/RNA- or protein-binding proteins (Figure 6A). This is not surprising as these proteins are largely involved in the control of transcription; an important regulatory activity expected in developing embryos. Furthermore proteins that regulate translation were found to constitute roughly 8%. Other major fractions of proteins detected were those that perform housekeeping functions such as catalysis (enzymes) (22%) and transport (17%).

We also determined the types metabolic pathways with which the kiwi genes are involved. This was done using information from the KEGG pathway database http://www.genome.jp/kegg/pathway.html for the chicken genes, which were then assigned to the corresponding orthologous kiwi genes. Figure 6B shows that roughly a quarter of kiwi genes were involved in genetic information processing pathways, which includes DNA replication and transcription. Approximately 27% and 21% of the genes were found to be associated with cellular and metabolic processes respectively. The remaining 14% of genes were shown to be involved in immunity and the endocrine system and 11% of the genes were associated with environmental processing including transport and signal transduction.

### Divergence times between Paleo- and Neognathae

**Figure 7:**
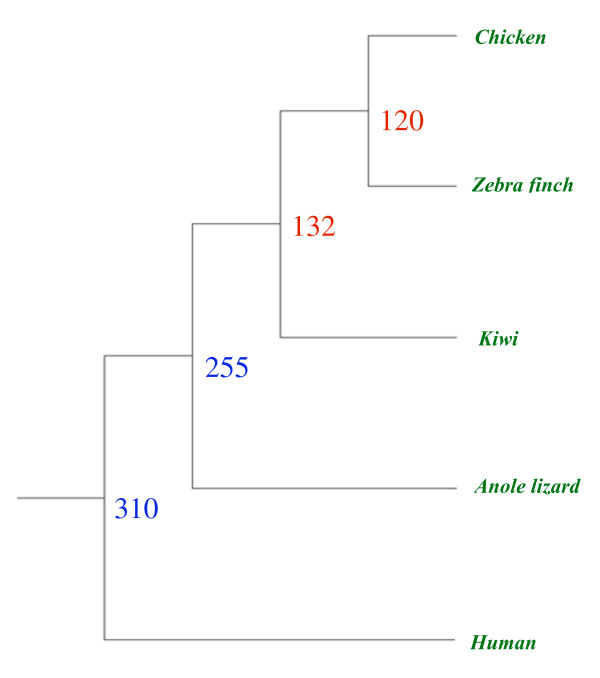
**Divergence times estimated using the software Multidivtime **[29]. Nuclear genes (702) from kiwi and other completed genomes of chicken, zebra finch, anole lizard, and human were used in this estimation. To calibrate the evolutionary divergences a fossil-based divergence time of 255 My [27] was used for the reptile-bird split.

We attempted to determine the divergence time between paleo- and neognathae birds as well as that between Galloanseriformes and the remaining neognathae birds. For this purpose we compared the genomes of neognathae birds (chicken and zebra finch), a reptile (anole lizard) and used a mammal (human) as an outgroup. The orthologous sequences of these genomes were obtained using reciprocal BLAST. This resulted in an orthologous dataset of 702 genes and included only those protein-coding sequences that align with the kiwi transcripts. The nucleotide sequences of all genes were concatenated and the branch lengths were estimated using BASEML under a general time reversible model of sequence evolution using the tree topology shown in Figure 7. First we conducted a simple direct estimation of the divergence times using pair-wise ML distances and a calibration time of 255 My (million years) between reptiles and birds. The latter was obtained from fossil studies [27]. This yielded a time of 130 My for the paleo- and neognathae split (using a kiwi and chicken/zebra finch pair) and 110 My for Galloanseriformes and other neognathae birds (chicken and zebra finch pair). Similar divergence times were obtained using a fossil calibration time of 310 My for the bird-mammal divergence [28]. We also performed a more rigorous Bayesian approach using the program Mutidivetime [29]. The divergence time estimates were 132 My (80 My - 170 My) for the paleo- and neognathae birds and 120 My (70 My - 150 My) for chicken-zebra finch pair, which are similar to those obtained using the direct method (Figure 7). Furthermore, we estimated the divergence times using BEAST without forcing any phylogenetic relationship among the species. Using the three birds and the anole lizard sequences we obtained the divergence times estimate by calibrating the molecular clock using the avian-reptile fossil based divergence time of 255 My. This analysis produced an estimate of 157 My (143 My -173 My), which is slightly higher than that obtained using the previous method. However, the confidence or HPD (Highest Posterior Density) intervals obtained from BEAST were within those obtained using Multidivtime. The divergence time computed by BEAST for the chicken-zebra finch split was 122 My (110 My-134 My), which is similar to that obtained using Multidivtime. Furthermore, we estimated the divergence times directly using the neutral synonymous evolutionary rates estimated by a previous study. A previous study using over 8,000 protein coding genes from chicken, zebra finch and anole lizard estimated the rate of neutral synonymous site evolution to be 1.23 × 10^-9 ^to 2.21 × 10^-9 ^[17]. Using an average rate of 1.7 × 10^-9 ^and a synonymous divergence of 0.452 we estimated the divergence time between kiwi (Paleognathae) and chicken (Neognathae) to be 133 My. Similarly using this rate and the neutral distance of 0.417, the divergence time for chicken-zebra finch pair was computed to be 123 My. Clearly the divergence times estimated in this study using the three different methods are largely similar. Furthermore these time estimates are comparable to those obtained using complete avian mitochondrial genomes [21,22].

## Conclusions

Using next generation sequencing technology our study provides some important insights into the conserved kiwi transcriptome. The neutral divergence at conserved protein coding genes of kiwi and chicken was found to be comparable to the synonymous divergence between human and mouse. However the divergence at amino acid replacement positions of these birds is much higher than the mammals suggesting a greater selective pressure in the latter. Similar to the observations from the studies on mammals, a negative relationship between gene expression levels and rate of protein evolution was found in birds. This study provides divergence time estimates between paleognathae and neognathae birds based on >700 nuclear genes.

The conserved kiwi transcriptome data reported here are useful for further specific studies on kiwi genetics and will assist future complete kiwi genome sequencing efforts, specifically in aiding genome assembly and determining gene structure. Importantly, our study provides a cost effective way to perform preliminary genome-based analyses and allows examination of some fundamental developmental and evolutionary processes of a species in the absence of a closely related genome.

## Methods

### Kiwi embryo

A young male kiwi embryo (sample k11-15) was kindly provided by Suzanne Bassett from the University of Otago, New Zealand. The embryo was void of any discernable structures and resembled an asymmetric gelatinous mass of approximately 15 mm in diameter. The small size and lack of obvious features suggested the embryo was at a very early stage of tissue building.

### RNA extraction and preliminary transcriptome characterization

Several 2 mm^2 ^slices were removed from equispaced regions around the kiwi embryo, combined, and total RNA was extracted using Trizol™, then precipitated with ethanol and resuspended in 50 μl of Milli-Q water. Five microlitres of total RNA was reverse transcribed in a 50 μl volume containing 50 mM Tris-Cl pH 8.8, 75 mM KCl, 5 mM MgCl_2_, 10 mM DTT, 100 ng oligodT_18 _0.5 mM of each dNTP, and 200 U of Moloney Murine Leukemia Virus reverse transcriptase. The mix was incubated at 42°C for 1 hr and extracted with phenol:chloroform. The complementary DNA (cDNA) was precipitated with ammonium acetate and ethanol, washed with 80% ethanol, and the resulting pellet was resuspended in 40 ul of H_2_O. Complementary DNA was amplified in 10 μl reactions containing 50 mM Tris-Cl pH 8.8, 20 mM (NH_4_)_2_SO_4_, 2.5 mM MgCl_2_, 1 mg/ml BSA, 200 uM of each dNTP, 40 ng of each primer, and ~0.3 U of platinum Taq (Invitrogen). The reaction mix was overlaid with mineral oil and subjected to amplification in a Hybaid OmniGene thermal cycler using the following parameters: 94°C for 2 min (× 1), 94°C for 20 sec, 54°C for 20 sec, 72°C for 20 sec (× 15), and then 94°C for 20 sec, 50°C for 20 sec, and 72°C for 20 sec (× 30). Amplified DNAs were detected by agarose gel electrophoresis in Tris-borate-EDTA buffer (TBE), stained with 50 ng/ml ethidium bromide in TBE, and then visualized over UV light. Positive amplifications were purified by centrifugation through ~40 μl of dry Sephacryl™S300HR and then sequenced at the Allan Wilson Centre Genome Sequencing Service using Applied Biosystems (ABI) BigDye^® ^Terminator v3.1 chemistry and an ABI3730 Genetic Analyzer. The primers used were designed to a selection of developmental and regulatory genes. In all cases the primers spanned an intron of at least 500 bp. The primers pairs used and genes targeted were: *tbx5*_2Fii- agtccaaagagctgcaggctga and *tbx5*_4R- catccgctggtacaatatccat; *cry1*F-tctgatgaccatgatgaga and *cry1*R-ctgtgtagaaaaattcacgcca; *px6*F-accatgcagaacagtcacag and *px6*R-acaacttcgggagtcgctact; *BMP4*F-tgctgcagatgtttgggct and *BMP4*R-ccgacgagatcacctcgtt; *ptx1*F-gccactttccagcggaaccg and *ptx1*R-gctcatggagttgaagaaggt; *hxB1*F-cggaccttcgattggatgaa and *hxB1*R-tcttgacttgggtttcgttgagct; *hxB8*F-caaatccaggagttctaccac and *hxB8*R-gtctggtagcggctgtaggt; *hxD12*F-tcaacttgaacctgacagt and *hxD12*R-cgtcggttctgaaaccaaatttt.

### Transcriptome preparation and amplification for FLX sequencing

Approximately 10 μg of total RNA was reverse transcribed using oligodT_18 _as outlined above, and second strand cDNA was synthesized in the same tube by adding 40 μl of 5× second strand buffer (100 mM Tris-Cl pH 7.0, 25 mM MgCl_2_, 450 mM KCl, 50 mM (NH_4_)_2_SO_4_), 4 μl of a 10 mM solution of each dNTP, 7 ul of 100 mM DTT, 20 U of *E. coli *DNA polymerase I, and water to 200 ul. The mix was incubated at room temp for 2 hrs, before the addition of 5 U of T4 DNA polymerase I to blunt the dsDNA ends, and the dsDNA was purified by phenol:chloroform extraction and ethanol precipitation. The dsDNA pellet was resuspended in 10 ul of 1 × Promega ligase buffer and then ligated together overnight at 4°C with 3 U of T4 DNA ligase. The ligated DNAs were purified and precipitated as described and resuspended in 10 μl of water. One microlitre of the DNA was then amplified using Templify (Amersham) as instructed by the manufacturer, and a sample of the amplified DNA was checked by gel electrophoresis. Approximately 2 μg of the amplified DNA was purified and sent to the University of Otago High-ThroughputDNA Sequencing Unit for megasequencing by FLX.

The amplified DNA was fragmented by nebulization. Sequencing adaptors were then ligated to the ends of these fragments and fragments that contained both adaptors were selected using biotin/streptavidin Library Immobilization Beads (Roche). The kiwi transcriptome library was not titrated. Instead an emPCR loading density of 1.5 copies per beads was chosen. Following this, the kiwi transcriptome library was annealed to enough DNA capture beads for 16 emulsions reactions of an emPCR I shotgun sequencing kit (Roche).

### Assembly of FLX sequence reads

The amplified kiwi cDNA library was prepared and sequenced using 454 FLX sequencing chemistry, which generated 75,632 sequence reads with an average length of 171 bp. These reads were used as queries to search a database of 22,000 chicken proteins downloaded from GenBank. We used BLASTX to translate the coding sequences into all six reading frames. Significant threshold levels were based on query protein length, as described before [30]. Homopolymer tracts and adaptors were removed using perl scripts as well as by manual examination. The number of kiwi reads that had significant hits with the chicken proteome were found to be 23,417 (31%). If there were more than two overlapping fragments the most frequent base was used to determine the consensus. These sequences were assembled using the criteria of a 20 bp identical overlap. Using blast2seq, chicken proteins and the translated segments were aligned and assembled. We used a stringent approach and extracted only the regions of kiwi coding sequences that had at least 90% identity to those of chicken. This conservative approach resulted in identification of 1,543 kiwi protein-coding genes with an average aligned (with chicken) length of 168 bp. This alignment did not show any bias in the genic location of the kiwi reads, as roughly 49% of the reads are from the 3' terminus of the genes and 51% are from the 5' terminus. Redundant (identical or subsets of) sequences were excluded from further analysis.

### Evolutionary rate estimation

The rate of evolution was estimated using conserved kiwi-chicken sequences. Sequence alignments from all 1,543 genes were concatenated and the divergences at synonymous and nonsynonymous sites were estimated using the pair-wise option of the CODEML program [31]. This approach was also followed for groups of genes such as highly expressed genes. Furthermore pair-wise likelihood distances at synonymous and nonsynonymous sites were also estimated for individual genes. Since the lengths of the sequences recovered were short (~168 bp) the dS estimates for individual genes are subjected to higher stochastic errors than the dN estimates, which might result in overestimation of the dN/dS ratios due to very small dS values. However we found only 10 genes with a very low dS (< 0.01) and therefore the genic dN/dS ratios obtained for most of the genes were not affected by this overestimation.

### Estimation of divergence times

Using a reciprocal BLAST hit approach [32] orthologous genes from three other vertebrates zebra finch, anole lizard, and human were also obtained. Protein sequences of five genomes (including chicken and kiwi) were aligned using CLUSTALW [33] and only those regions that aligned with the partial kiwi proteins were extracted. cDNA sequences of all these genomes were aligned using the protein alignments as a guide. The resultant 702 genes from five genomes were concatenated. Since the phylogenetic relationship between these five species is well known, the tree topology (as in Figure 7) was used to obtain the branch lengths using the program BASEML from PAML [31]. For this analysis a GTR+gamma (five categories) model was used.

To estimate divergence times we followed a Bayesian based approach implemented in the software Multidivtime [29,34]. The molecular clock was calibrated using the well documented fossil-based estimate of 255 My (252 My - 257 My) for the reptile-avian split [27] and the human sequence was used as an outgroup. The lower and upper constraints used in the program were 230 My and 280 My. We used 255 My as the expected time between the (ingroup) root to the tip (rttime). The prior rate was calculated as the ratio of the median of the branch lengths from root-to-tip and the time elapsed as per the suggestion given in the documentation (Thorne and Kishino 2002). The prior standard deviation was kept as 50 My. Other priors used were as outlined in the Multidivtime documentation. Furthermore we used BEAST [35] to estimate the divergence times without constraining any phylogenic relationship among the species. For this purpose we used three birds and the lizard protein coding sequences. We used the Tamura Nei +Gamma model for sequence evolution and the reptile-bird fossil based divergence time to calibrate the molecular clock.

## Authors' contributions

DML and CDM conceived the study. LH conducted laboratory experiments. SS performed data analysis and wrote the paper. DML, LH and CDM edited the paper. All authors read and approved the manuscript.

## References

[B1] Fleischmann RD Adams MD White O Clayton RA Kirkness EF Kerlavage AR Bult CJ Tomb JF Dougherty BA Merrick JM Mckenney K Sutton G Fitzhugh W Fields C Gocayne JD Scott J Shirley R Liu LI Glodek A Kelley JM Weidman JF Phillips CA Spriggs T Hedblom E Cotton MD Utterback TR Hanna MC Nguyen DT Saudek DM Brandon RC Fine LD Fritchman JL Fuhrmann JL Geoghagen NSM Gnehm CL Mcdonald LA Small KV Fraser CM Smith HO Venter JC Whole-Genome Random Sequencing and Assembly of Haemophilus-Influenzae Rd Science 1995 269 496 512 10.1126/science.7542800 7542800

[B2] Lander ES Linton LM Birren B Nusbaum C Zody MC Baldwin J Devon K Dewar K Doyle M FitzHugh W Funke R Gage D Harris K Heaford A Howland J Kann L Lehoczky J LeVine R McEwan P McKernan K Meldrim J Mesirov JP Miranda C Morris W Naylor J Raymond C Rosetti M Santos R Sheridan A Sougnez C Stange-Thomann N Stojanovic N Subramanian A Wyman D Rogers J Sulston J Ainscough R Beck S Bentley D Burton J Clee C Carter N Coulson A Deadman R Deloukas P Dunham A Dunham I Durbin R French L Grafham D Gregory S Hubbard T Humphray S Hunt A Jones M Lloyd C McMurray A Matthews L Mercer S Milne S Mullikin JC Mungall A Plumb R Ross M Shownkeen R Sims S Waterston RH Wilson RK Hillier LW McPherson JD Marra MA Mardis ER Fulton LA Chinwalla AT Pepin KH Gish WR Chissoe SL Wendl MC Delehaunty KD Miner TL Delehaunty A Kramer JB Cook LL Fulton RS Johnson DL Minx PJ Clifton SW Hawkins T Branscomb E Predki P Richardson P Wenning S Slezak T Doggett N Cheng JF Olsen A Lucas S Elkin C Uberbacher E Frazier M Gibbs RA Muzny DM Scherer SE Bouck JB Sodergren EJ Worley KC Rives CM Gorrell JH Metzker ML Naylor SL Kucherlapati RS Nelson DL Weinstock GM Sakaki Y Fujiyama A Hattori M Yada T Toyoda A Itoh T Kawagoe C Watanabe H Totoki Y Taylor T Weissenbach J Heilig R Saurin W Artiguenave F Brottier P Bruls T Pelletier E Robert C Wincker P Rosenthal A Platzer M Nyakatura G Taudien S Rump A Yang HM Yu J Wang J Huang GY Gu J Hood L Rowen L Madan A Qin SZ Davis RW Federspiel NA Abola AP Proctor MJ Myers RM Schmutz J Dickson M Grimwood J Cox DR Olson MV Kaul R Raymond C Shimizu N Kawasaki K Minoshima S Evans GA Athanasiou M Schultz R Roe BA Chen F Pan HQ Ramser J Lehrach H Reinhardt R McCombie WR de la Bastide M Dedhia N Blocker H Hornischer K Nordsiek G Agarwala R Aravind L Bailey JA Bateman A Batzoglou S Birney E Bork P Brown DG Burge CB Cerutti L Chen HC Church D Clamp M Copley RR Doerks T Eddy SR Eichler EE Furey TS Galagan J Gilbert JGR Harmon C Hayashizaki Y Haussler D Hermjakob H Hokamp K Jang WH Johnson LS Jones TA Kasif S Kaspryzk A Kennedy S Kent WJ Kitts P Koonin EV Korf I Kulp D Lancet D Lowe TM McLysaght A Mikkelsen T Moran JV Mulder N Pollara VJ Ponting CP Schuler G Schultz JR Slater G Smit AFA Stupka E Szustakowki J Thierry-Mieg D Thierry-Mieg J Wagner L Wallis J Wheeler R Williams A Wolf YI Wolfe KH Yang SP Yeh RF Collins F Guyer MS Peterson J Felsenfeld A Wetterstrand KA Patrinos A Morgan MJ Conso IHGS Initial sequencing and analysis of the human genome Nature 2001 409 860 921 10.1038/35057062 11237011

[B3] Mardis ER Next-generation DNA sequencing methods Annu Rev Genomics Hum Genet 2008 9 387 402 10.1146/annurev.genom.9.081307.164359 18576944

[B4] Shendure J Ji HL Next-generation DNA sequencing Nat Biotechnol 2008 26 1135 1145 10.1038/nbt1486 18846087

[B5] Bentley DR Balasubramanian S Swerdlow HP Smith GP Milton J Brown CG Hall KP Evers DJ Barnes CL Bignell HR Boutell JM Bryant J Carter RJ Cheetham RK Cox AJ Ellis DJ Flatbush MR Gormley NA Humphray SJ Irving LJ Karbelashvili MS Kirk SM Li H Liu XH Maisinger KS Murray LJ Obradovic B Ost T Parkinson ML Pratt MR Rasolonjatovo IMJ Reed MT Rigatti R Rodighiero C Ross MT Sabot A Sankar SV Scally A Schroth GP Smith ME Smith VP Spiridou A Torrance PE Tzonev SS Vermaas EH Walter K Wu XL Zhang L Alam MD Anastasi C Aniebo IC Bailey DMD Bancarz IR Banerjee S Barbour SG Baybayan PA Benoit VA Benson KF Bevis C Black PJ Boodhun A Brennan JS Bridgham JA Brown RC Brown AA Buermann DH Bundu AA Burrows JC Carter NP Castillo N Catenazzi MCE Chang S Cooley RN Crake NR Dada OO Diakoumakos KD Dominguez-Fernandez B Earnshaw DJ Egbujor UC Elmore DW Etchin SS Ewan MR Fedurco M Fraser LJ Fajardo KVF Furey WS George D Gietzen KJ Goddard CP Golda GS Granieri PA Green DE Gustafson DL Hansen NF Harnish K Haudenschild CD Heyer NI Hims MM Ho JT Horgan AM Hoschler K Hurwitz S Ivanov DV Johnson MQ James T Jones TAH Kang GD Kerelska TH Kersey AD Khrebtukova I Kindwall AP Kingsbury Z Kokko-Gonzales PI Kumar A Laurent MA Lawley CT Lee SE Lee X Liao AK Loch JA Lok M Luo SJ Mammen RM Martin JW McCauley PG McNitt P Mehta P Moon KW Mullens JW Newington T Ning ZM Ng BL Novo SM O'Neill MJ Osborne MA Osnowski A Ostadan O Paraschos LL Pickering L Pike AC Pike AC Pinkard DC Pliskin DP Podhasky J Quijano VJ Raczy C Rae VH Rawlings SR Rodriguez AC Roe PM Rogers J Bacigalupo MCR Romanov N Romieu A Roth RK Rourke NJ Ruediger ST Rusman E Sanches-Kuiper RM Schenker MR Seoane JM Shaw RJ Shiver MK Short SW Sizto NL Sluis JP Smith MA Sohna JES Spence EJ Stevens K Sutton N Szajkowski L Tregidgo CL Turcatti G vandeVondele S Verhovsky Y Virk SM Wakelin S Walcott GC Wang JW Worsley GJ Yan JY Yau L Zuerlein M Rogers J Mullikin JC Hurles ME McCooke NJ West JS Oaks FL Lundberg PL Klenerman D Durbin R Smith AJ Accurate whole human genome sequencing using reversible terminator chemistry Nature 2008 456 53 59 10.1038/nature07517 18987734 PMC2581791

[B6] Green RE Krause J Ptak SE Briggs AW Ronan MT Simons JF Du L Egholm M Rothberg JM Paunovic M Paabo S Analysis of one million base pairs of Neanderthal DNA Nature 2006 444 330 336 10.1038/nature05336 17108958

[B7] Poinar HN Schwarz C Qi J Shapiro B MacPhee RDE Buigues B Tikhonov A Huson DH Tomsho LP Auch A Rampp M Miller W Schuster SC Metagenomics to paleogenomics: Large-scale sequencing of mammoth DNA Science 2006 311 392 394 10.1126/science.1123360 16368896

[B8] Zerbino DR Birney E Velvet: Algorithms for de novo short read assembly using de Bruijn graphs Genome Res 2008 18 821 829 10.1101/gr.074492.107 18349386 PMC2336801

[B9] Li RQ Fan W Tian G Zhu HM He L Cai J Huang QF Cai QL Li B Bai YQ Zhang ZH Zhang YP Wang W Li J Wei FW Li H Jian M Li JW Zhang ZL Nielsen R Li DW Gu WJ Yang ZT Xuan ZL Ryder OA Leung FCC Zhou Y Cao JJ Sun X Fu YG Fang XD Guo XS Wang B Hou R Shen FJ Mu B Ni PX Lin RM Qian WB Wang GD Yu C Nie WH Wang JH Wu ZG Liang HQ Min JM Wu Q Cheng SF Ruan J Wang MW Shi ZB Wen M Liu BH Ren XL Zheng HS Dong D Cook K Shan G Zhang H Kosiol C Xie XY Lu ZH Zheng HC Li YR Steiner CC Lam TTY Lin SY Zhang QH Li GQ Tian J Gong TM Liu HD Zhang DJ Fang L Ye C Zhang JB Hu WB Xu AL Ren YY Zhang GJ Bruford MW Li QB Ma LJ Guo YR An N Hu YJ Zheng Y Shi YY Li ZQ Liu Q Chen YL Zhao J Qu N Zhao SC Tian F Wang XL Wang HY Xu LZ Liu X Vinar T Wang YJ Lam TW Yiu SM Liu SP Zhang HM Li DS Huang Y Wang X Yang GH Jiang Z Wang JY Qin N Li L Li JX Bolund L Kristiansen K Wong GKS Olson M Zhang XQ Li SG Yang HM Wang J Wang J The sequence and de novo assembly of the giant panda genome Nature 2010 463 311 317 10.1038/nature08696 20010809 PMC3951497

[B10] Hillier LW Miller W Birney E Warren W Hardison RC Ponting CP Bork P Burt DW Groenen MAM Delany ME Dodgson JB Chinwalla AT Cliften PF Clifton SW Delehaunty KD Fronick C Fulton RS Graves TA Kremitzki C Layman D Magrini V McPherson JD Miner TL Minx P Nash WE Nhan MN Nelson JO Oddy LG Pohl CS Randall-Maher J Smith SM Wallis JW Yang SP Romanov MN Rondelli CM Paton B Smith J Morrice D Daniels L Tempest HG Robertson L Masabanda JS Griffin DK Vignal A Fillon V Jacobbson L Kerje S Andersson L Crooijmans RPM Aerts J van der Poel JJ Ellegren H Caldwell RB Hubbard SJ Grafham DV Kierzek AM McLaren SR Overton IM Arakawa H Beattie KJ Bezzubov Y Boardman PE Bonfield JK Croning MDR Davies RM Francis MD Humphray SJ Scott CE Taylor RG Tickle C Brown WRA Rogers J Buerstedde JM Wilson SA Stubbs L Ovcharenko I Gordon L Lucas S Miller MM Inoko H Shiina T Kaufman J Salomonsen J Skjoedt K Wong GKS Wang J Liu B Wang J Yu J Yang HM Nefedov M Koriabine M deJong PJ Goodstadt L Webber C Dickens NJ Letunic I Suyama M Torrents D von Mering C Zdobnov EM Makova K Nekrutenko A Elnitski L Eswara P King DC Yang S Tyekucheva S Radakrishnan A Harris RS Chiaromonte F Taylor J He JB Rijnkels M Griffiths-Jones S Ureta-Vidal A Hoffman MM Severin J Searle SMJ Law AS Speed D Waddington D Cheng Z Tuzun E Eichler E Bao ZR Flicek P Shteynberg DD Brent MR Bye JM Huckle EJ Chatterji S Dewey C Pachter L Kouranov A Mourelatos Z Hatzigeorgiou AG Paterson AH Ivarie R Brandstrom M Axelsson E Backstrom N Berlin S Webster MT Pourquie O Reymond A Ucla C Antonarakis SE Long MY Emerson JJ Betran E Dupanloup I Kaessmann H Hinrichs AS Bejerano G Furey TS Harte RA Raney B Siepel A Kent WJ Haussler D Eyras E Castelo R Abril JF Castellano S Camara F Parra G Guigo R Bourque G Tesler G Pevzner PA Smit A Fulton LA Mardis ER Wilson RK Sequence and comparative analysis of the chicken genome provide unique perspectives on vertebrate evolution Nature 2004 432 695 716 10.1038/nature03154 15592404

[B11] Axelsson E Smith NGC Sundstrom H Berlin S Ellegren H Male-biased mutation rate and divergence in autosomal, Z-linked and W-linked introns of chicken and turkey Mol Biol Evol 2004 21 1538 1547 10.1093/molbev/msh157 15140948

[B12] Axelsson E Webster MT Smith NGC Burt DW Ellegren H Comparison of the chicken and turkey genomes reveals a higher rate of nucleotide divergence on microchromosomes than macrochromosomes Genome Res 2005 15 120 125 10.1101/gr.3021305 15590944 PMC540272

[B13] Warren WC Clayton DF Ellegren H Arnold AP Hillier LW Kunstner A Searle S White S Vilella AJ Fairley S Heger A Kong L Ponting CP Jarvis ED Mello CV Minx P Lovell P Velho TA Ferris M Balakrishnan CN Sinha S Blatti C London SE Li Y Lin YC George J Sweedler J Southey B Gunaratne P Watson M Nam K Backstrom N Smeds L Nabholz B Itoh Y Whitney O Pfenning AR Howard J Volker M Skinner BM Griffin DK Ye L McLaren WM Flicek P Quesada V Velasco G Lopez-Otin C Puente XS Olender T Lancet D Smit AF Hubley R Konkel MK Walker JA Batzer MA Gu W Pollock DD Chen L Cheng Z Eichler EE Stapley J Slate J Ekblom R Birkhead T Burke T Burt D Scharff C Adam I Richard H Sultan M Soldatov A Lehrach H Edwards SV Yang SP Li X Graves T Fulton L Nelson J Chinwalla A Hou S Mardis ER Wilson RK The genome of a songbird Nature 2010 464 757 762 10.1038/nature08819 20360741 PMC3187626

[B14] Axelsson E Ellegren H Quantification of Adaptive Evolution of Genes Expressed in Avian Brain and the Population Size Effect on the Efficacy of Selection Mol Biol Evol 2009 26 1073 1079 10.1093/molbev/msp019 19188264

[B15] Axelsson E Hultin-Rosenberg L Brandstrom M Zwahlen M Clayton DF Ellegren H Natural selection in avian protein-coding genes expressed in brain Mol Ecol 2008 17 3008 3017 10.1111/j.1365-294X.2008.03795.x 18482257

[B16] Ekblom R Balakrishnan CN Burke T Slate J Digital gene expression analysis of the zebra finch genome BMC Genomics 2010 11 219 10.1186/1471-2164-11-219 20359325 PMC2996964

[B17] Nam K Mugal C Nabholz B Schielzeth H Wolf JB Backstrom N Kunstner A Balakrishnan CN Heger A Ponting CP Clayton DF Ellegren H Molecular evolution of genes in avian genomes Genome Biol 2010 11 R68 10.1186/gb-2010-11-6-r68 20573239 PMC2911116

[B18] Barker MS Dlugosch KM Reddy ACC Amyotte SN Rieseberg LH SCARF: maximizing next-generation EST assemblies for evolutionary and population genomic analyses Bioinformatics 2009 25 535 536 10.1093/bioinformatics/btp011 19129211

[B19] Kunstner A Wolf JBW Backstrom N Whitney O Balakrishnan CN Day L Edwards SV Janes DE Schlinger BA Wilson RK Jarvis ED Warren WC Ellegren H Comparative genomics based on massive parallel transcriptome sequencing reveals patterns of substitution and selection across 10 bird species Mol Ecol 2010 19 266 276 10.1111/j.1365-294X.2009.04487.x 20331785 PMC2904817

[B20] Kumar S Hedges SB A molecular timescale for vertebrate evolution Nature 1998 392 917 920 10.1038/31927 9582070

[B21] Brown JW Rest JS Garcia-Moreno J Sorenson MD Mindell DP Strong mitochondrial DNA support for a Cretaceous origin of modern avian lineages BMC Biol 2008 6 6 18226223 10.1186/1741-7007-6-6 PMC2267772

[B22] Pereira SL Baker AJ A mitogenomic timescale for birds detects variable phylogenetic rates of molecular evolution and refutes the standard molecular clock Mol Biol Evol 2006 23 1731 1740 10.1093/molbev/msl038 16774978

[B23] Benton MJ Donoghue PC Paleontological evidence to date the tree of life Mol Biol Evol 2007 24 26 53 10.1093/molbev/msl150 17047029

[B24] Pal C Papp B Hurst LD Highly expressed genes in yeast evolve slowly Genetics 2001 158 927 931 11430355 10.1093/genetics/158.2.927 PMC1461684

[B25] Subramanian S Kumar S Gene expression intensity shapes evolutionary rates of the proteins encoded by the vertebrate genome Genetics 2004 168 373 381 10.1534/genetics.104.028944 15454550 PMC1448110

[B26] Subramanian S Nearly neutrality and the evolution of codon usage bias in eukaryotic genomes Genetics 2008 178 2429 2432 10.1534/genetics.107.086405 18430960 PMC2323827

[B27] Reisz RR Muller J Molecular timescales and the fossil record: a paleontological perspective Trends Genet 2004 20 237 241 10.1016/j.tig.2004.03.007 15109777

[B28] Benton MJ The fossil record2 1993 London: Chapman and Hall, London

[B29] Thorne JL MULIDISTRIBUTE 2003 http://statgen.ncsu.edu/thorne/multidivtime.html

[B30] Duret L Mouchiroud D Gouy M Hovergen - a Database of Homologous Vertebrate Genes Nucleic Acids Res 1994 22 2360 2365 10.1093/nar/22.12.2360 8036164 PMC523695

[B31] Yang ZH PAML 4: Phylogenetic analysis by maximum likelihood Mol Biol Evol 2007 24 1586 1591 10.1093/molbev/msm088 17483113

[B32] Altschul SF Madden TL Schaffer AA Zhang JH Zhang Z Miller W Lipman DJ Gapped BLAST and PSI-BLAST: a new generation of protein database search programs Nucleic Acids Res 1997 25 3389 3402 10.1093/nar/25.17.3389 9254694 PMC146917

[B33] Larkin MA Blackshields G Brown NP Chenna R McGettigan PA McWilliam H Valentin F Wallace IM Wilm A Lopez R Thompson JD Gibson TJ Higgins DG Clustal W and clustal X version 2.0 Bioinformatics 2007 23 2947 2948 10.1093/bioinformatics/btm404 17846036

[B34] Thorne JL Kishino H Divergence time and evolutionary rate estimation with multilocus data Syst Biol 2002 51 689 702 10.1080/10635150290102456 12396584

[B35] Drummond AJ Rambaut A BEAST: Bayesian evolutionary analysis by sampling trees BMC Evol Biol 2007 7 214 10.1186/1471-2148-7-214 17996036 PMC2247476

